# Association of depression with evolution of heart failure in patients with type 2 diabetes mellitus

**DOI:** 10.1186/s12933-018-0664-5

**Published:** 2018-01-24

**Authors:** Ying Wang, Hong Yang, Mark Nolan, John Burgess, Kazuaki Negishi, Thomas H. Marwick

**Affiliations:** 1 Menzies Institute for Medical Research, Baker Heart and Diabetes Institute, Melbourne, Australia; 2 Faculty of Health Sciences, Baker Heart and Diabetes Institute, Melbourne, Australia; 3 Baker Heart and Diabetes Institute, 75 Commercial Road, Melbourne, VIC 3004 Australia

**Keywords:** Depression, Incident heart failure, Type 2 diabetes mellitus

## Abstract

**Background:**

Depression is a prevalent, independent predictor of mortality in patients with heart failure (HF). Depression is also common in type 2 diabetes mellitus (T2DM), which is itself an important risk factor for HF. However, association of depression with incident HF in T2DM is undefined. The aim of the present study was to evaluate the predictive value of depression in predicting incident HF in a community-based cohort of asymptomatic patients with T2DM.

**Methods:**

We prospectively recruited 274 asymptomatic T2DM patients ≥ 65 years (age 71 ± 4 year, 56% men) with preserved EF and no ischemic heart disease from a community-based population. The Patient Health Questionnaire 9 (PHQ-9) was used to detect depression, and LV dysfunction was sought with a comprehensive echocardiogram, including LV hypertrophy (LVH) and subclinical diastolic function (E/e′). Over a median follow-up of 1.5 years (range 0.5–3), 20 patients were lost to follow-up and 254 individuals were followed for outcomes.

**Results:**

At baseline, depression was present in 9.5%, LVH was identified in 26% and reduced E/e′ in 11%. Over a median follow-up of 1.5 years, 37 of 245 patients developed new-onset HF and 3 died, giving an event rate of 107/1000 person-years. In a competing-risks regression analysis, depression (adjusted HR 2.54, 95% CI 1.18–5.46; p = 0.017) was associated with incident HF and had incremental predictive power to clinical, biochemical and echocardiographic variables.

**Conclusion:**

Depression is prevalent in asymptomatic elderly patients with T2DM, and depression independently and incrementally predicts incident HF.

## Background

Heart failure (HF) has been recognized as one of the most common and malignant complications of type 2 diabetes mellitus (T2DM) [1]. HF has been reported in > 22% elderly patients with diabetes, and the HF incidence rate was 12.6 per 100 person-years [2]. Among elderly patients with DM, the 5-year mortality rate was approximately ninefold higher in those who developed HF than in those who did not [2]. Depressive symptoms are common in the community, but the 19% prevalence of depression in patients with T2DM is about double that of those without DM [3]. In patients with existing HF, comorbid depression and diabetes are associated with a higher mortality and rehospitalisation rate [4]. The presence of depression more than doubled the risk of all-cause of mortality in HF (hazard ratio [HR] 2.29; 95% CI 0.94–5.40; p = 0.05), and both DM and depression led to a nearly fourfold increment in all-cause mortality (HR 3.71; 95% CI 1.49–9.25; p = 0.005) [4].

Depression is an independent predictor of HF onset in elderly patients with isolated systolic hypertension, which is independent of demographic characteristics, medical history and myocardial infarction risks [5]. Although prior studies have found the association of depression and adverse outcomes with diabetes alone or heart failure alone, the association of depression with incident HF in patients with diabetes is unclear, especially as death is a competing risk. The current study was undertaken to evaluate the extent to which level of depression is able to identify risk of incident HF in in a community-based cohort of asymptomatic patients with T2DM.

## Methods

### Study population

We prospectively recruited 274 asymptomatic T2DM patients aged ≥ 65 with preserved LVEF from a community-based population in Australia. Patients with existing HF or known ischemic heart disease (reported with coronary artery disease including CABG and/or myocardial infarction with regional scar) were excluded, as were patients with more than moderate valve disease, history of HF, LVEF < 40%, or inability to acquire adequate echocardiographic images for baseline analysis.

### Ethics, consent and permissions

All participants provided written, informed consent, and the study protocol was approved by the Tasmanian Human Research Ethics Committee.

### Clinical features

T2DM was based on self-report of this diagnosis including the use of relevant medication. Obesity was defined as body mass index (BMI) ≥ 30 kg/m^2^. Demographics, disease and family history and medication use were obtained using a standardized questionnaire. BMI was calculated as weight in kilograms divided by height in meters squared. In addition to standardized weight and height measurements, waist circumference (WC) at the midpoint between the lower costal margin and the iliac crest was measured to the nearest millimeter by a trained examiner. After at least 10 min rest in a quiet room, supine resting blood pressure (BP) was measured twice and averaged in each patient. Active hypertension was defined by a mean systolic blood pressure (BP) ≥ 140 mmHg or a diastolic BP ≥ 90 mmHg [6]. International Federation of Clinical Chemistry (IFCC) standardized hemoglobin A1c (cutoff 64 mmol/mol), fasting glucose, creatinine and estimated glomerular filtration rate (eGFR) were extracted from pathology records. To estimate missing values for HbA1c, we carried out imputation using linear regression equation.

### Depression assessment

All participants completed the Patient Health Questionnaire 9 (PHQ-9) at baseline assessment. Each item in the PHQ-9 questionnaire corresponds to one of the nine DSM-IV criteria for diagnosis of major depression, and respondents indicate their level of agreement with each item (0 = not at all, 1 = several days, 2 = more than half of the days, 3 = nearly every day). The validated cutoff PHQ-9 score ≥ 10 was used as diagnosis for the presence of depression [7], and further stratified respondents into four categories of depressive symptomatology based on the total PHQ-9 score: minimal (0–4), mild (5–9), moderate (10–20), and severe (≥ 20).

### Echocardiography

A comprehensive echocardiogram including standard transthoracic 2D and Doppler echocardiography was performed using the same ultrasound machine (Siemens ACUSON SC2000, 4V1c and 4Z1c probes, Siemens Healthcare, Mountain View, CA) in accordance with the American Society of Echocardiography guidelines [8, 9]. Images were saved in raw data format and analyzed offline. LV internal dimensions and wall thickness, chamber volumes and valvular morphology were assessed. LV mass index (LVMi) was obtained from LV mass measurement using standard criteria and normalized for body size (body surface area or height to the power of 1.7). LV hypertrophy (LVH) was defined as LVMi (normalized for body surface area) > 115 g/m^2^ for males and > 95 g/m^2^ for females. LVEF was measured using the modified Simpson’s biplane method. LV inflow was obtained using pulsed wave Doppler in the apical 4-chamber view; peak early (E) and late (A) diastolic velocities, deceleration time and E/A ratio were assessed. Peak early diastolic medial and lateral mitral annular velocity (e′) and the ratio of mitral inflow early diastolic velocity to average e′ velocity were obtained from pulsed tissue Doppler; E/e′ > 13 was used as an indicator of diastolic dysfunction [10].

### Outcomes

The primary endpoint was new-onset of HF, and all-cause mortality was considered as a competing risk. Potential HF symptoms were assessed through regular follow-up phone calls, followed by symptom surveillance questionnaires, clinical visits and repeated echocardiography. Records of all-cause hospitalization and mortality were obtained from administrative data. The diagnosis of HF was established according to Framingham HF criteria; symptoms and physical signs that were suspicious for HF were reviewed by three independent cardiologists [11].

### Statistical analysis

Descriptive data are presented as mean ± standard deviation (SD) and dichotomous data as subject number and percentage. Comparisons between the groups were performed by independent samples t test; the Kruskal–Wallis test was used for comparison of non-normally distributed variables. Univariable and multivariable stepwise forward linear regression were performed in order to identify the variables with significant association with PHQ-9 score.

Univariable Cox regression was used to identify the predictors of incident HF among clinical, demographic and echocardiographic variables. We fitted a competing-risk model to compute hazard ratio (HR) and 95% confidence interval (95% CI) for the associations between each risk factor and incident HF [12]. A multivariable model was constructed to determine the independent predictors, guided by univariable analyses and the clinical relevance of the variables. Competing risk methods were used to account for the competing risk of death when analyzing the endpoint of time to HF. The cumulative incidences of HF were calculated and graphically displayed separately for patients with and without depression. Gray’s K-sample test was to compare the cumulative incidence estimates of HF between patients with/without depression [13], which accounts for all-cause of death as a competing risk of HF. All data were analyzed using standard statistical computer software (SPSS 22, IBM, Chicago, IL and Stata, V.12.0, StataCorp, College Station, Texas, USA); p < 0.05 was deemed to be statistically significant.

## Results

### Patient characteristics

Table 1 describes the clinical, echocardiographic and biochemical features of 274 asymptomatic T2DM patients ≥ 65 years old with preserved EF from the community who were prospectively recruited and underwent baseline tests. Table 1 also includes the 254 T2DM participants who were included in the final analysis (see below). In the total recruited group of 274 subjects, the prevalence of LV dysfunction was 13% (by LVH) and 11% (by abnormal E/e′ cutoff 13). In the 254 subjects included in the final analysis, baseline HbA1c was available in 196 individuals (age 71 ± 4 years, 55% men). In this subgroup, the baseline HbA1c was 53.2 ± 11.4 mmol/mol. After imputation of missing values, the baseline HbA1c was 53.5 ± 10.1 mmol/mol (Table 1).

**Table 1:** Baseline characteristics (demographic, clinical, echocardiographic, physiologic) of 274 elderly asymptomatic patients with T2DM

Demographic and clinical characteristics	
Age (years)	71 ± 4.4
Male gender (n, %)	150 (54.7)
Weight (kg)	85.8 ± 17.2
Height (cm)	168.4 ± 10.0
BMI (kg/m^2^)	30.3 ± 5.9
Waist circumference (cm)	103.2 ± 13.3
Obesity (n, %)	135 (49.3)
Heart rate (n/min)	69 ± 11
Systolic blood pressure (mmHg)	139 ± 15
Diastolic blood pressure (mmHg)	81 ± 10
Hypertension (n, %)	204 (74.4)
Family history of HF (n, %)	80 (29.2)
Past chemotherapy (n, %)	25 (9.1)
Past heart disease (n, %)	16 (5.8)
Medication	
Insulin (n, %)	65 (23.7)
Metformin (n, %)	184 (67.2)
ACEi/ARB (n, %)	183 (66.8)
Beta-blockers (n, %)	15 (5.5)
Calcium antagonists (n, %)	64 (23.4)
Diuretics (n, %)	31 (11.3)
Lipid lowering meds (n, %)	133 (48.5)
Questionnaire	
PHQ-9 score	3.2 ± 4.3
0–4	210 (76.7)
5–9	37 (13.5)
10–20	25 (9.1)
≥ 20	2 (0.7)
Echocardiography	
LV ejection fraction (%)	63.1 ± 6.4
Mitral early-diastolic inflow velocity (E wave) (m/s)	0.65 ± 0.17
Mitral late-diastolic inflow velocity (A wave) (m/s)	0.83 ± 0.19
Transmitral diastolic flow velocity ratio (E/A)	0.79 ± 0.22
Early diastolic mitral annular velocity (e′) (m/s)	0.08 ± 0.02
Mitral E/e′ septal–lateral ratio (E/e′)	9.2 ± 2.8
E/e′ ratio > 13 (n, %)	29 (10.6)
Deceleration time (DT) (s)	246.8 ± 52.4
LV mass index (g/m^2^)	85.7 ± 19.0
LV mass index (g/m^1.7^)	74.4 ± 20.8
LV hypertrophy (n, %)^a^	36 (13.2)
Biochemical characteristics	
HbA1c (mmol/mol) (n = 274)	53.5 ± 10.1
Fasting glucose (µmol/L) (n = 109)	8.3 ± 3.5
Creatinine (µmol/L) (n = 186)	81.3 ± 24.0
eGFR (mL/min/1.73 m^2^) (n = 187)	
≥ 90	37 (19.8)
60–89	115 (61.5)
45–59	25 (13.4)
30–44	9 (4.8)
15–29	1 (0.5)

^a^LV hypertrophy was defined as LVMi > 115 g/m^2^ for males, > 95 g/m^2^ for female

### Depression

Based on PHQ-9 score, 37 (14%) patients had minimal-to-mild depressive symptoms (PHQ-9 score 5–9), and 25 (9%) patients were identified as having moderate-to-severe depressive symptoms (PHQ-9 score 10–20), and 2 (0.7%) patients were identified as having severe depressive symptoms (PHQ-9 score ≥ 20), and were diagnosed as having depression using the cutoff of PHQ-9 score ≥ 10. Table 2 shows the features associated with depression; these patients had higher BMI and more central obesity, more insulin use and higher HbA1c level. However, there was no difference in age, gender, blood pressure, heart rate and other risk factors of HF and echocardiographic parameters among patients with and without depression. The only independent determinant of PHQ-9 score detected by linear regression was BMI.

**Table 2:** Baseline demographic, clinical and echocardiographic variable comparisons among T2DM patients categorized by the presence of depression (n = 254)

	Depression (n = 25)	No depression (n = 229)	p
Demographic and clinical characteristics			
Age (years)	70.6 ± 3.8	70.9 ± 4.4	0.418
Male gender (n, %)	11 (44.0)	131 (57.2)	0.208
Weight (kg)	92.6 ± 22.8	85.1 ± 16.5	0.122
BMI (kg/m^2^)	34.1 ± 7.8	29.8 ± 5.6	*0.012*
Waist circumference (cm)	109.0 ± 14.2	102.4 ± 13.1	*0.032*
Obesity (n, %)	18 (72.0)	106 (46.3)	*0.015*
Heart rate (n/min)	70 ± 11	68 ± 11	0.280
Systolic blood pressure (mmHg)	138 ± 17	139 ± 14	0.936
Diastolic blood pressure (mmHg)	80 ± 12	81 ± 9	0.748
Hypertension (n, %)	19 (76.0)	171 (74.7)	0.985
Family history of HF (n, %)	10 (40.0)	63 (27.5)	0.191
Past chemotherapy (n, %)	4 (16.0)	18 (7.9)	0.170
Past heart disease (n, %)	3 (12.0)	11 (4.8)	0.135
HbA1c (mmol/mol)	57.6 ± 10.6	53.0 ± 10.0	*0.049*
HbA1c > 64 mmol/mol (n, %)	8 (32)	24 (10.5)	*0.002*
Medication			
Insulin (n, %)	11 (44.0)	49 (21.4)	*0.012*
Metformin (n, %)	18 (72.0)	157 (68.6)	0.725
ACEi/ARB (n, %)	19 (76.0)	152 (66.4)	0.331
Beta-blockers (n, %)	1 (4.0)	14 (6.1)	0.571
Calcium antagonists (n, %)	7 (28.0)	54 (23.6)	0.524
Diuretics (n, %)	4 (16.0)	23 (10.0)	0.360
Lipid lowering meds (n, %)	14 (56.0)	109 (47.5)	0.426
Echocardiography			
LV ejection fraction (%)	61.5 ± 8.7	63.2 ± 6.2	0.222
Mitral early-diastolic inflow velocity (E wave) (m/s)	0.62 ± 0.18	0.65 ± 0.16	0.530
Mitral late-diastolic inflow velocity (A wave) (m/s)	0.81 ± 0.16	0.83 ± 0.19	0.525
Transmitral diastolic flow velocity ratio (E/A)	0.79 ± 0.21	0.77 ± 0.25	0.693
Early diastolic mitral annular velocity (e′) (m/s)	0.07 ± 0.01	0.08 ± 0.02	0.852
Mitral E/e′ septal–lateral ratio (E/e′)	8.6 ± 1.9	9.2 ± 2.8	0.336
E/e′ ratio > 13 (n, %)	1 (4.0)	25 (10.9)	0.280
Deceleration time (DT) (s)	231.6 ± 50.4	248.6 ± 52.9	0.129
LV mass index (g/m^2^)	87.3 ± 16.1	85.4 ± 19.6	0.602
LV mass index (g/m^1.7^)	82.3 ± 27.0	73.6 ± 20.2	0.130
LV hypertrophy (n, %)^a^	2 (8.0)	33 (14.4)	0.998

Italic values indicate significant associations

^a^LV hypertrophy was defined as LVMi > 115 g/m^2^ for males, > 95 g/m^2^ for females

### Follow-up

**Fig. 1:**
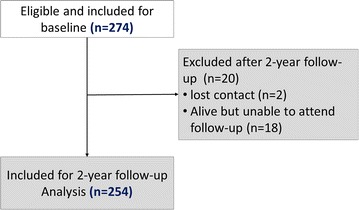
Flow chart of study inclusion (n = 254)

After a median follow-up time of 1.5 years (range 0.5–3 years), 2 of 274 T2DM participants (0.7%) were lost to follow-up and 18 of 274 participants (6.6%) were alive but unable to attend for clinic review (Fig. 1). This group was no different from the remaining 254 individuals who completed follow-up (Appendix 1: Table 5). The primary composite endpoint was reached by 40 patients; 37 patients developed new-onset HF and 3 died, giving an event rate of 107/1000 person-years.

Cox regression analysis was performed to analyze the association between clinical and biochemical variables and echocardiographic parameters of interest and the time to the primary composite endpoint (Table 3). Patients with events were characterized by obesity, worse glycemic control, baseline LVEF, LV mass (but not diastolic function parameters), beta blockade and depression. PHQ-9 scores were significantly associated with events, both as a continuous or binomial covariate. When PHQ-9 score was categorized into three levels (0–4, 5–9 and ≥ 10), PHQ-9 ≥ 10 was independently associated with events. The cumulative survival of the included 254 elderly asymptomatic T2DM stratified by the severity of depression is shown in Appendix 3: Fig. 4. The cumulative survival for patients with PHQ-9 score ≥ 10 was the lowest. A multivariable Cox regression model (informed by significant univariable clinical associations and echocardiographic parameters of interest) was constructed to determine the independent predictors of the composite endpoint (Table 4). Obesity, LVH and depression were associated with increased risks of the composite endpoint.

**Table 3:** Univariable Cox regression analysis for primary composite endpoint in 254 elderly asymptomatic patients with T2DM

	HR (95% CI)	p value
Demographic and clinical characteristics		
Age	1.06 (0.99, 1.32)	0.064
Male gender (yes/no)	1.81 (0.92, 3.56)	0.086
Weight	1.04 (1.02, 1.05)	*<* *0.001*
BMI	1.10 (1.06, 1.15)	*<* *0.001*
Waist circumference	1.05 (1.03, 1.08)	*<* *0.001*
Obesity (yes/no)	3.61 (1.76, 7.39)	*<* *0.001*
Heart rate	0.99 (0.97, 1.03)	0.727
Systolic blood pressure	0.99 (0.97, 1.01)	0.314
Diastolic blood pressure	0.90 (0.99, 1.03)	0.895
Hypertension (yes/no)	1.07 (0.52, 2.19)	0.854
Family history of HF (yes/no)	0.70 (0.33, 1.47)	0.349
Past chemotherapy (yes/no)	1.54 (0.60, 3.93)	0.536
Past heart disease (yes/no)	1.46 (0.45, 4.74)	0.529
HbA1c (mmol/mol)	1.03 (1.01, 1.06)	*0.009*
HbA1c > 64 mmol/mol (n, %)	3.27 (1.66, 6.43)	*0.001*
Medication		
Insulin (yes/no)	1.77 (0.92, 3.39)	0.086
Metformin (yes/no)	0.72 (0.38, 1.36)	0.310
ACEi/ARB (yes/no)	1.12 (0.57, 2.20)	0.749
Beta-blockers (yes/no)	3.25 (1.36, 7.77)	*0.008*
Calcium antagonists (yes/no)	0.50 (0.21, 1.18)	0.114
Diuretics (yes/no)	0.91 (0.33, 2.65)	0.909
Lipid lowering med (yes/no)	1.32 (0.71, 2.48)	0.382
Questionnaire		
PHQ-9 score (continuous)	1.08 (1.01, 1.16)	*0.021*
PHQ-9 score ≥ 10 (yes/no)	3.21 (1.41, 7.30)	*0.005*
PHQ-9 score (categorical)		
0–4	Ref	
5–9	0.58 (0.18, 1.89)	0.362
≥ 10	3.01 (1.32, 6.89)	*0.009*
Echocardiography		
LV ejection fraction	0.95 (0.90, 0.99)	*0.018*
Mitral early-diastolic inflow velocity (E wave)	3.46 (0.54, 22.39)	0.192
Mitral late-diastolic inflow velocity (A wave)	1.81 (0.32, 10.31)	0.503
Transmitral diastolic flow velocity ratio (E/A)	0.92 (0.16, 5.31)	0.929
Early diastolic mitral annular velocity (e′)	1.05 (0.86, 1.28)	0.644
Mitral E/e′ septal–lateral ratio	1.05 (0.94, 1.17)	0.437
E/e′ ratio > 13 (yes/no)	1.04 (0.37, 2.94)	0.934
Deceleration time (DT)	0.99 (0.99, 1.00)	0.363
LV mass index (g/m^2^)	1.03 (1.02, 1.04)	*<* *0.001*
LV mass index (g/m^1.7^)	1.03 (1.02, 1.04)	*<* *0.001*
LV hypertrophy (yes/no)^a^	3.92 (2.04, 7.52)	*<* *0.001*

Italic values indicate significant associations

^a^LV hypertrophy was defined as LVMi > 115 g/m^2^ for males, > 95 g/m^2^ for females

**Table 4:** Cox regression analysis for primary composite endpoint in 254 elderly asymptomatic patients with T2DM

Variable	No. of patients	No. of HF	No. of death	Unadjusted HR (95% CI) p value	LVH-adjusted HR (95% CI)^a^ p value	E/e′-adjusted HR (95% CI)^b^ p value	Adjusted HR (95% CI)^c^ p value
Age	254	37	3	1.06 (0.99, 1.13) 0.064	–	–	1.06 (0.99, 1.14) 0.055
Male gender	142	25	3	1.81 (0.92, 3.56) 0.086	–	–	1.36 (0.61, 2.92) 0.466
Obesity	124	29	1	3.61 (1.76, 7.39) *<* *0.001*	2.87 (1.38, 5.98) *0.005*	3.27 (1.58, 5.75) *0.001*	2.97 (1.44, 6.30) *0.004*
HbA1c	32	10	2	3.27 (1.66, 6.43) *0.001*	2.04 (0.99, 4.22) 0.054	2.47 (1.22, 5.00) *0.012*	2.01 (0.93, 4.10) 0.077
LVH	35	13	1	3.92 (2.04, 7.52) *<* *0.001*	3.24 (1.65, 6.38) *0.001*	–	2.67 (1.25, 5.99) *0.011*
E/e′	26	4	0	1.04 (0.37, 2.94) 0.934	–	1.11 (0.39, 3.18) 0.845	0.82 (0.26, 2.52) 0.724
Depression	25	7	0	3.21 (1.41, 7.30) *0.005*	2.80 (1.16, 6.76) *0.022*	2.39 (1.01, 5.67) *0.048*	3.14 (1.27, 7.74) *0.013*

Obesity, BMI ≥ 30 kg/m^2^; poor HbA1c, ICFFstd-HbA1c > 64 mmol/mol; LVH, left ventricular hypertrophy, cutoff > 115 g/m^2^ for males, > 95 g/m^2^ for females; E/e′ ratio, cutoff > 13; depression, the score of PHQ-9 questionnaire ≥ 10

*HR* hazard ratio, *CI* confidence interval

Italic values indicate significant associations

^a^In the multivariate cox model, obesity, poor HbA1c, LVH and depression were entered into the model

^b^In the multivariate cox model, obesity, poor HbA1c, E/e′ and depression were entered into the model

^c^In the multivariate cox model, age, male gender, obesity, poor HbA1c, LVH, E/e′ and depression were entered into the model

### Prediction of HF in T2DM

**Fig. 2:**
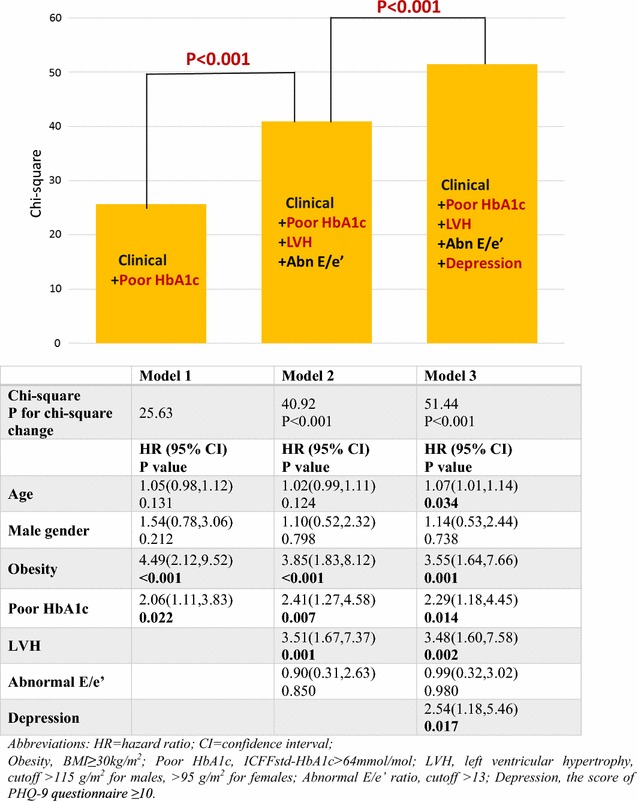
Prediction of incident HF in elderly asymptomatic patients with T2DM (competing risk analysis). LVH and depression showed incremental value over clinical parameters for incident HF

A competing risk analysis that controlled for age, obesity and poor glycemic control was performed to assess whether depression and echocardiographic parameters of interest were associated with incident HF in elderly asymptomatic patients with T2DM (Fig. 2). The baseline clinical model, showing an independent effect of obesity (p < 0.001), and poor glycemic control (p = 0.02) on incident events, was improved by addition of echocardiographic features (LVH, p = 0.001). However, the addition of depression (p = 0.017) had incremental predictive power to clinical, biochemical and echocardiographic variables for the prediction of incident HF (Fig. 2).

**Fig. 3:**
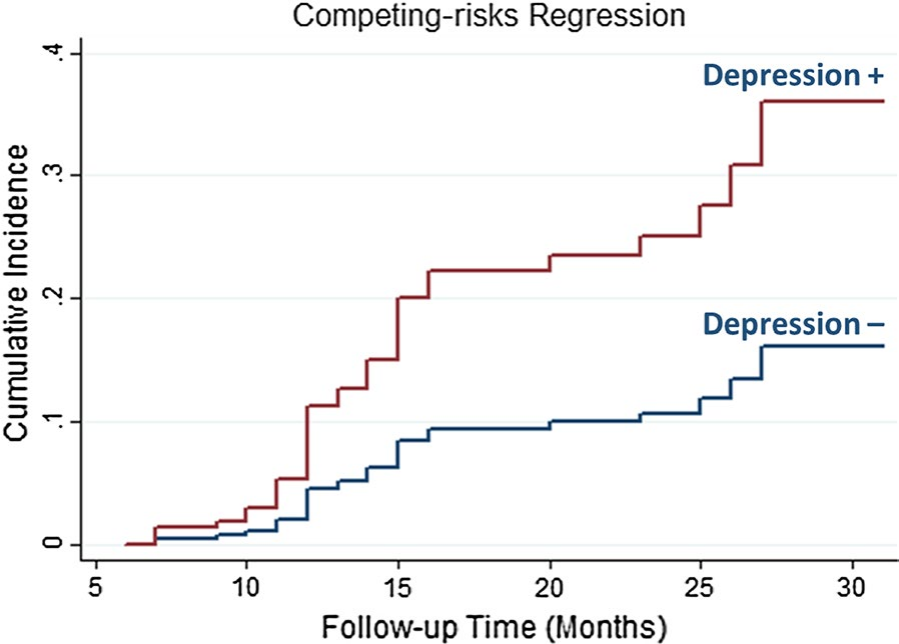
Cumulative incidence estimates of HF in elderly asymptomatic T2DM stratified by patients with and without depression

**Fig. 4:**
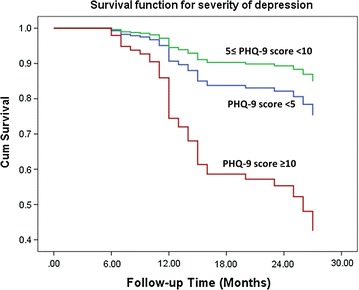
Cumulative survival of 254 elderly asymptomatic T2DM stratified by severity of depression

The cumulative incidence of HF with time among elderly asymptomatic T2DM stratified by depression status is shown in Fig. 3. By the end of the follow-up, considering the competing risk, the cumulative incidence of HF was 0.36 in patients with depression and was 0.15 in patients without depression. Gray’s test also showed that the cumulative incidence of HF was significantly lower in patients with depression than those patients without depression (p < 0.001).

## Discussion

The results of this study show that depression (defined as PHQ-9 score ≥ 10) was significantly associated with increased risk of incident HF during follow-up of asymptomatic elderly patients with T2DM and preserved EF. This association was independent of clinical factors (including age, gender and BMI) and echocardiographic features such as LV hypertrophy and diastolic function. The presence of depression increased the likelihood of incident HF by 2.5-fold, and increased the composite endpoint 3.1-fold, compared with those without depression. Although depression has been linked with adverse outcomes in HF patients, to our knowledge, this is the first study to show an independent association between depression and incident HF in DM.

### Depression and HF

Depression is a common co-morbidity of HF, with variable reports of its prevalence. A meta-analysis of 25 studies showed depression to be present in 11% HF in NYHA (New York Heart Association) functional class I, 20% with class II, and approximately 40% of class III and IV HF. Depression is more widely diagnosed when this diagnosis is made by questionnaire (34%), and less with clinical interview (19%) [14].

Depression is an independent predictor of adverse clinical outcomes in HF, and increases the risk of mortality by 40–50% [15] In elderly patients with newly diagnosed HF, the presence of depression increased the risk of hospital admission by 20%, and depression screening has been suggested as a routine assessment at the time of HF diagnosis [16]. Even mild depression (defined as PHQ-9 score ≥ 5) has been associated with mortality and re-hospitalization in HF [17]. Depression is also associated with a 2.4-fold increment of mortality, independent of age, gender, etiology, NYHA class, EF and LV systolic dysfunction [18].

Few previous studies have sought whether psychosocial factors could independently predict incident HF [5, 19, 20]. Abramson et al. [5] first found that depression, as defined by Center for Epidemiological Studies Depression Scale (CES-D) ≥ 16, was independently associated with a substantial increase in the risk of HF among elderly patients with isolated systolic hypertension. Williams et al. [20] found an independent association between depression (CES-D ≥ 20, and incident HF among elderly women but not elderly men. The results from prospective cohort studies are contradictory—the Nord-Trøndelag Health (HUNT) Study demonstrated the prospective association of self-reported depression at baseline with future HF in a dose–response manner, independent of a large number of baseline cardiovascular risk factors, acute myocardial infarction (AMI) and several chronic disorders [19]. However, the Multi-Ethnic Study of Atherosclerosis (MESA) found no significant association between depression (CES-D scale ≥ 16), and subsequent HF in an older population. However, in participants reporting fair/poor health status at baseline, this association was significant [21]. It is certainly plausible that sympathetic overactivity might be associated with both HF mortality and HF incidence, but this remains unproven.

### Depression and diabetes

About 20–30% of elderly patients with diabetes suffer from clinical depression, and around 10% of them have major depression [22]. Even though the relationship between depression and diabetes is incompletely understood, it is clear that depression has an adverse impact on the course of diabetes, and diabetes complications may result in both the risk of depression and worsening the course of depression. Their association is primarily driven by somatic-affective symptoms of depression [23]. Additionally, the duration of depression lasts longer (usually ≥ 2 years) in most diabetic patients and the relapse rate is relatively high [24]. A previous study with 10-year follow-up also showed that all-cause of mortality increased with the severity of depression in diabetic subjects [25].

### HF in diabetes

Numerous epidemiological studies have indicated the strong association between diabetes and the development of HF [2, 26]. The underlying mechanisms are multiple, but there is a growing recognition of a primary myocardial disease process—“diabetic cardiomyopathy”—that may lead to LV dysfunction, and subsequently HF. Hyperinsulinemia, inflammation and oxidative stress result in increased fatty acid metabolism and fetal gene expression [27]. The resulting pathophysiological changes include impaired myocardial relaxation and cardiomyocyte resting tension, activation of the renin–angiotensin system (leading to vasoconstriction, salt and water retention, and fibrosis) and diabetic autonomic neuropathy. These changes lead to impaired diastolic and systolic function [28]. Furthermore, overweight has been proved to have a greater effect on LV function in diabetes than in non-diabetes [29]. In our study, obesity has emerged as an important association of incident HF.

Several shared pathophysiological mechanisms may link T2DM, depressive symptoms and incident HF. Pro-inflammatory cytokines and platelet activation, rhythm disturbances, neurohormonal activation, endothelial dysfunction and hypercoagulability are present in patients with depression [30]. These pathophysiological changes adversely influence the cardiovascular system and have been postulated to play an important role in the development and progression of HF [30]. Endothelial dysfunction plays an important role in cardiovascular homeostasis by producing various vascular regulators that mediate fibrinolysis, hypercoagulability, platelet activation and vascular tone, disturbances of which constitute the step linking diabetes to cardiovascular events [31]. Hyperglycemia and insulin resistance are associated with endothelial dysfunction [32] and cardiac damage [31]. Impaired nitric oxide production and oxidative stress may also lead to endothelial dysfunction, impaired vasodilation and large vessel impairment [31]. Hyperglycemia and hyperinsulinemia have also been significant contributors to cardiovascular complications through their role in stimulating coagulation and impairing fibrinolysis [33]. Pro-inflammatory cytokines are common in diabetes and significantly increase the risk of progression of cardiovascular complications at all stages [34]. Additionally, patients with depression are more likely to take up risky lifestyles and behaviors and more likely to show non-adherence to medical regimens and behavior recommendations that affects the prognosis of HF [35]. Depression may be also involved with the lower social support that is implicated in the development of HF.

### Limitation

In the present study, the population were selected from the community with preserved EF, so diabetes patients with established asymptomatic LV systolic dysfunction were excluded. We did not have data on brain natriuretic peptide (BNP). Although this biomarker has an established role in the acute care setting to assist the diagnosis of congestive heart failure and in predicting long-term outcomes, its utility for the early detection of HF with preserved ejection fraction (HFpEF) is more controversial. Its response to raised filling pressure is blunted in overweight patients compared with normal weight patients, regardless of the presence or absence of cardiovascular disease [36].

We used self-reported measures of symptoms of depression rather than diagnostic interviews. However, the use of the PHQ-9 survey for screening depression has been validated and has been recommended by the American Heart Association for the diagnosis of depression in coronary heart disease [37]. We acknowledge that the lower sensitivity of PHQ-9 relative to other markers [38] means that it may not capture all patients. However, its high specificity can identify patients at high risk for adverse cardiovascular events. While the study shows a connection of depression with LV dysfunction, it does not explain the reasons for depression. In our study, the factor associated with depression was advanced BMI. Previous research has demonstrated the association of diabetes-related complications, longer duration of diabetes, more demanding regimens, inactivity, current smoking and overweight as potential risk factors for depression in T2DM [39].

While we excluded patients with reported history of CAD including CABG and/or AMI with regional scar, testing for occult CAD was not possible in this community-based study. It is therefore possible that some of the abnormal cardiac function signal is attributable to coronary artery disease. In addition, the blood examination in the cohort such as BNP or NT-pro BNP were not part of this study. Lastly, despite the longitudinal study design and high-risk nature of this population, the number of events was relatively small.

## Conclusion

In this community cohort of asymptomatic patients with T2DM, depression was prevalent, and significantly associated with incident HF over 2-years of observation. This association is not explained by baseline demographic characteristics, glycemic control and LV dysfunction [including LV hypotrophy and subclinical diastolic dysfunction (evidenced by E/e′)]. The mechanism of this association requires further investigation, and although it remains unclear as to whether a depression intervention in T2DM may prevent HF, depressed patients may warrant closer monitoring for the development of HF.

## Appendices

### Appendix 1

See Table 5.

**Table 5:** Comparison of baseline characteristics between patients who completed follow-up study (n = 254) and patients who loss to follow-up study (n = 20)

	Completed n = 254	Loss to FU n = 20	p value
Demographic and clinical characteristics			
Age (years)	70.9 ± 4.3	72 ± 5.1	0.341
Male gender (n, %)	142 (55.9)	8 (40.0)	0.389
Weight (kg)	85.8 ± 17.3	85.7 ± 16.7	0.820
BMI (kg/m^2^)	30.2 ± 5.9	31.1 ± 5.6	0.536
Waist circumference (cm)	103.1 ± 13.3	107.3 ± 15.3	0.255
Obesity (n, %)	124 (48.8)	11 (55.0)	0.811
Heart rate (n/min)	69 ± 11	71 ± 13	0.445
Systolic blood pressure (mmHg)	139 ± 14	141 ± 23	0.550
Diastolic blood pressure (mmHg)	81 ± 10	80 ± 10	0.613
Hypertension (n, %)	190 (74.8)	14 (70.0)	0.195
Family history of HF (n, %)	73 (28.7)	7 (35.0)	0.518
Past chemotherapy (n, %)	22 (8.7)	3 (15.0)	0.403
Past heart disease (n, %)	14 (5.5)	2 (10.0)	0.103
HbA1c (mmol/mol)	53.4 ± 10.2	54.2 ± 9.5	0.733
HbA1c > 64 mmol/mol (n, %)	32 (12.6)	4 (20)	0.245
Medication			
Insulin (n, %)	60 (23.6)	5 (25.0)	*0.037*
Metformin (n, %)	175 (68.9)	9 (45.0)	0.244
ACEi/ARB (n, %)	171 (67.3)	12 (60.0)	0.630
Beta-blockers (n, %)	15 (5.9)	0 (0)	*<* *0.001*
Calcium antagonists (n, %)	62 (24.3)	2 (10.0)	0.093
Diuretics (n, %)	27 (10.6)	4 (20.0)	0.445
Lipid lowering meds (n, %)	123 (48.4)	10 (50.0)	0.560
Questionnaire			
PHQ-9 score	3.2 ± 4.3	2.8 ± 5.1	0.787
0–4	194 (76.4)	16 (80.0)	0.508
5–9	35 (13.8)	2 (10.0)	0.633
10–20	24 (9.4)	1 (5.0)	0.185
≥ 20	1 (0.4)	1 (5.0)	*0.011*
Echocardiography			
LVEF (%)	63.1 ± 6.5	63.0 ± 5.1	0.965
E wave (m/s)	0.65 ± 0.16	0.74 ± 0.26	*0.024*
A wave (m/s)	0.83 ± 0.19	0.88 ± 0.25	0.421
E/A ratio	0.79 ± 0.21	0.79 ± 0.28	0.974
e′ (m/s)	0.08 ± 0.02	0.08 ± 0.02	0.512
E/e′ ratio	9.2 ± 2.7	9.8 ± 3.1	0.347
E/e′ ratio > 13 (n, %)	26 (10.2)	3 (15.0)	0.086
DT (s)	247.0 ± 52.8	244.6 ± 50.7	0.844
LV mass index (g/m^2^)	85.6 ± 19.3	86.9 ± 15.4	0.735
LV mass index (g/m^1.7^)	74.5 ± 21.1	73.3 ± 17.2	0.775
LV hypertrophy (n, %)^a^	35 (13.8)	1 (5.9)	0.354

Italic values indicate significant associations

^a^LV hypertrophy was defined as LVMi > 115 g/m^2^ for males, > 95 g/m^2^ for females

### Appendix 2

See Table 6.

**Table 6:** Multivariate linear regression model for prediction of PHQ-9 score in older asymptomatic T2DM

Chi square 0.043	Β (95% CI)	p value
Age	− 0.04 (− 5.45, 12.08)	0.457
Gender	− 0.97 (− 2.00, 0.05)	0.063
BMI	0.12 (0.03, 0.20)	*0.009*

Italic values indicate significant associations

### Appendix 3

See Fig. 4.

## Abbreviations

BMI: body mass index
BP: blood pressure
E/e′: ratio of transmitral flow to annular tissue velocity
e′: early diastolic medial and lateral mitral annular velocity
eGFR: estimated glomerular filtration rate
LVEF: ejection fraction
LVH: LV hypertrophy
HF: heart failure
HR: hazard ratio
LV: left ventricular
LVMi: LV mass index
PHQ-9: Patient Health Questionnaire 9
T2DM: type 2 diabetes mellitus
WC: waist circumference
